# Supplementary material for: Prognostic effect of pretreatment albumin-to-alkaline phosphatase ratio in human cancers: A meta-analysis
Source: PLoS One. 2020 Aug 21;15(8):e0237793. doi: 10.1371/journal.pone.0237793 (PMC7444501; doi:10.1371/journal.pone.0237793)
Supplement: S2 Table — (DOCX) [file pone.0237793.s002.docx]

| #1 | Add | Search ((((tumor[Title/Abstract]) OR neoplasms[Title/Abstract]) OR cancer[Title/Abstract]) OR carcinoma[Title/Abstract]) OR malignancy[Title/Abstract] |
| --- | --- | --- |
| #2 | Add | Search (((albumin/alkaline phosphatase ratio[Title/Abstract]) OR albumin to alkaline phosphatase[Title/Abstract]) OR albumin to alkaline phosphatase ratio[Title/Abstract]) OR AAPR[Title/Abstract] |
| #3 | Add | Search ((prognosis[Title/Abstract]) OR prognostic[Title/Abstract]) OR survival[Title/Abstract] |
| #4 | Add | Search(((((((tumor[Title/Abstract]) OR neoplasms[Title/Abstract]) OR cancer[Title/Abstract]) OR carcinoma[Title/Abstract]) OR malignancy[Title/Abstract])) AND ((((albumin/alkaline phosphatase ratio[Title/Abstract]) OR albumin to alkaline phosphatase[Title/Abstract]) OR albumin to alkaline phosphatase ratio[Title/Abstract])) OR AAPR[Title/Abstract] AND (((prognosis[Title/Abstract]) OR prognostic[Title/Abstract]) OR survival[Title/Abstract]) |

**Literature search strategy in PUBMED**
