# Supplementary material for: Prognostic effect of pretreatment albumin-to-alkaline phosphatase ratio in human cancers: A meta-analysis
Source: PLoS One. 2020 Aug 21;15(8):e0237793. doi: 10.1371/journal.pone.0237793 (PMC7444501; doi:10.1371/journal.pone.0237793)
Supplement: S3 Table — (DOCX) [file pone.0237793.s003.docx]

**S3 Table. Quality assessment of all included studies according to the Newcastle-Ottawa Scale.**

| **Study** | **Selection** | | | | **Comparability** | **Outcome** | | |
| --- | --- | --- | --- | --- | --- | --- | --- | --- |
|  | **Representativeness of exposed** | **Selection of non-exposed** | **Ascertainment of exposure** | **Outcome of interest was not present at start of study** |  | **Assessment of outcome** | **Duration of follow-up** | **Adequacy of follow-up** |
| Li SJ *et al.* | * | * | * | * | ** | * | * | * |
| Li D *et al.* | * | * | * | * | ** | * |  | * |
| Li XG *et al.* | * | * | * | * | * | * |  | * |
| Zhang *et al.* | * | * | * | * | ** | * | * | * |
| Nie *et al.* | * | * | * | * | ** | * | * | * |
| Kim *et al.* | * | * | * | * | ** |  | * | * |
| Chan *et al.*  (training) | * | * | * | * | ** | * |  | * |
| Chan *et al.*  (validation) | * | * | * | * | ** | * | * | * |
| Cai *et al.* | * | * | * | * | * | * |  | * |
| Chen *et al.*  (training) | * | * | * | * | ** | * |  | * |
| Chen *et al.*  (validation1) | * | * | * | * | ** | * |  | * |
| Chen *et al.*  (validation2) | * | * |  | * | ** | * |  | * |
| Xiong *et al.* | * | * | * | * | ** | * | * | * |
| Long *et al.* | * | * | * | * | ** | * |  | * |
| Tan *et al.* | * | * | * | * | ** | * | * | * |
| Xia *et al.* | * | * | * | * | ** | * | * | * |
