# Supplementary figures and images for: Prognostic effect of pretreatment albumin-to-alkaline phosphatase ratio in human cancers: A meta-analysis
Source: PLoS One. 2020 Aug 21;15(8):e0237793. doi: 10.1371/journal.pone.0237793 (PMC7444501; doi:10.1371/journal.pone.0237793)

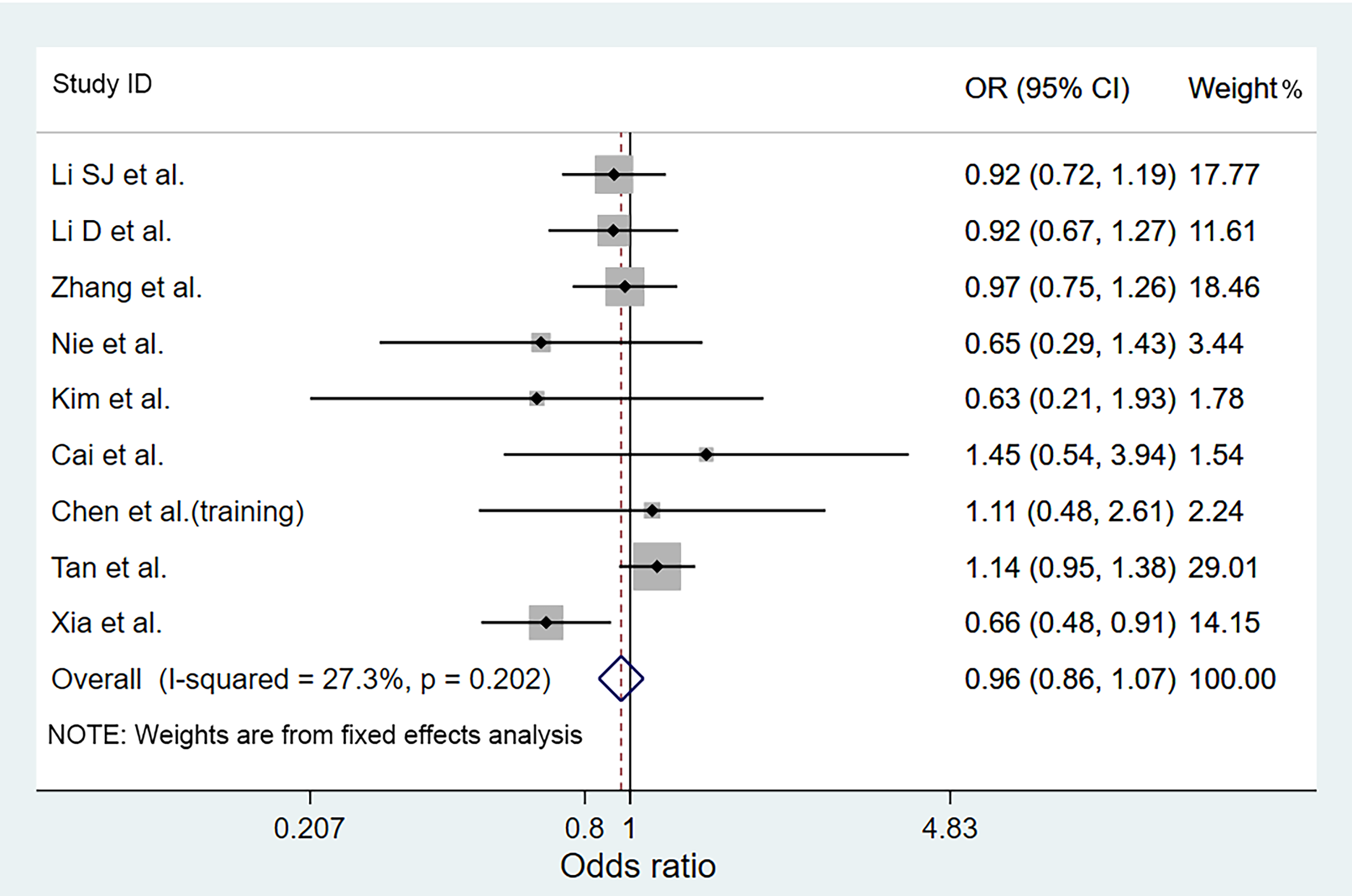

Supplement: S1 Fig — (TIF) [file pone.0237793.s004.tif]

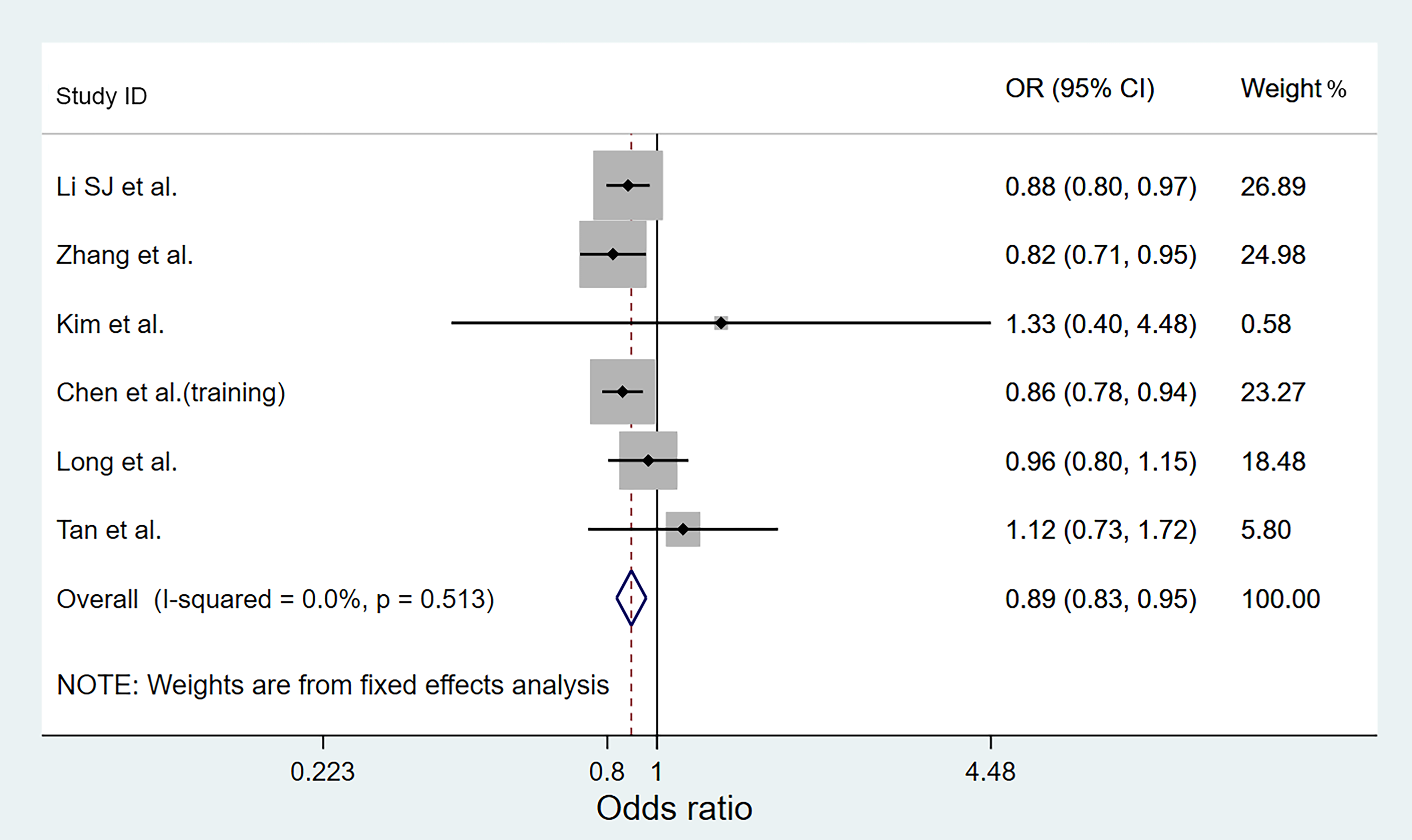

Supplement: S2 Fig — (TIF) [file pone.0237793.s005.tif]
